# Uptake of ^3^H-cAMP by retinal pigment epithelium isolated from bluegill sunfish (*Lepomis macrochirus*)

**DOI:** 10.1186/1471-2202-7-82

**Published:** 2006-12-29

**Authors:** Thomas A Keith, Varsha Radhakrishnan, Steve Moredock, Dana M García

**Affiliations:** 1 Department of Biology, Texas State University-San Marcos, San Marcos, Texas 78666, USA

## Abstract

**Background:**

In bluegill sunfish, the melanin-containing pigment granules of the retinal pigment epithelium undergo cyclic movements in response both to ambient lighting and circadian cues. Pigment granules aggregate into the cell body at night (in the dark), and disperse into apical processes during the day (in the light). Regulation of pigment granule aggregation in a number of fishes depends on modulating the intracellular levels of cyclic adenosine monophosphate.

**Results:**

Here we show isolated RPE takes up cyclic adenosine monophosphate (cAMP) in a saturable manner, exogenously applied cAMP induces pigment granule aggregation in retinal pigment epithelium isolated from bluegill, and aggregation induced in this manner is inhibited by treatment with probenecid, an organic anion transport inhibitor.

**Conclusion:**

Our results raise the possibility that cAMP functions as a messenger secreted from the neural retina to signal darkness to the RPE, which takes it up. It further suggests that organic anion transport systems are the route by which cAMP crosses RPE cell membranes since probenecid inhibits extracellular cAMP from causing pigment granule aggregation.

## Background

In the eyes of teleosts and many other vertebrates, the retinal photoreceptors (rods and cones) and the retinal pigment epithelium (RPE) interact continuously during normal physiological conditions to optimize light capture for vision. The RPE lies posterior and adjacent to the retinal photoreceptors; therefore, light entering the eye through the pupil strikes the photoreceptors before the RPE. The apical surface of RPE is directly adjacent to the photoreceptor outer segments, while the basal surface lies next to the choroid coat. From the apical surface of RPE, delicate apical processes interdigitate with the photoreceptors. In green sunfish (*Lepomis cyanellus*) the apical projections are often greater than 100 μm long [1]

Pigment granules inside the RPE cells migrate into the apical projections from the cell body to shade the rods when ambient light increases in intensity or as a response to circadian cues [2]. The movements of the pigment granules are one element of retinomotor movements; additional elements include cell shape changes manifested by rod and cone photoreceptors [3]. In teleosts and other lower vertebrates, pupils are often fixed in diameter; thus, instead of pupillary constriction, the coordinated movements of the photoreceptors and RPE pigment granules are thought to regulate the amount of light reaching the retinal photoreceptors [4]. The shading effect brought about by dispersion of the pigment granules into the apical processes prevents excessive photobleaching of the rods in bright light. This protection, in turn, ensures that rod photoreceptors retain sensitivity during dim light conditions [2]. During dark conditions pigment granules aggregate into the RPE cell body [3,5].

The retina must communicate with the RPE in order for coordinated retinomotor movements to occur. Communication depends on chemical messengers that diffuse across the subretinal space from the neural retina to the RPE [6]. This hypothesis is supported by studies showing that light does not stimulate pigment granule movement in isolated RPE cells [1,7]. The subretinal space is known to contain an abundance of neurotransmitters and messenger molecules. For example, dopamine is released from the retina when changes in ambient light turn from dark to light [6]. Dopamine binds D2 receptors on the RPE cell membrane and subsequently induces light-adaptive pigment granule dispersion [7].

The classical second messenger cyclic adenosine monophosphate (cAMP) has been found to induce dark-adaptive pigment granule aggregation in isolated RPE cells [1,8], and dark-adapted eyes from a number of species are known to contain elevated levels of cAMP [9-12]. The actual mechanism by which cAMP induces pigment granule aggregation is not yet known, but presumably it works through a cAMP-dependent protein kinase, as is the case in melanophores [13]. Forskolin, a stimulator of adenylyl cyclase, has also been found to mimic dark adaptation by inducing pigment granule aggregation in isolated RPE cells [1,8]. Thus, both intracellularly synthesized cAMP and exogenous cAMP added to the incubation medium are capable of inducing pigment granule aggregation.

Two possible mechanisms by which exogenous cAMP affects RPE are (1) by crossing the membrane via organic anion transporter mechanisms or (2) by acting on membrane receptors to stimulate adenylyl cyclase. García and Burnside's [8] results using organic anion transport inhibitors support the hypothesis that cAMP enters RPE isolated from green sunfish (*Lepomis cyanellus*) via organic anion transport proteins. According to the model they proposed, cAMP could efflux from retinal cells in the dark and accumulate in the subretinal space. The cAMP molecules in the subretinal space would then enter RPE cells via organic anion transporters and induce pigment granule aggregation [8].

The study presented here was designed to determine whether cAMP was taken up by isolated RPE and to compare the effects of cAMP and organic anion transport inhibitors on RPE isolated from bluegill sunfish (*Lepomis macrochirus*) to published effects on green sunfish RPE [1,8]. We found that RPE takes up cAMP, that non-derivatized cAMP induces pigment granule aggregation with maximal aggregation achieved by doses of 1 mM cAMP, that cAMP-induced aggregation was blocked by the organic anion transport inhibitor probenecid. Our results extend those presented by García and Burnside [8] using green sunfish by demonstrating cAMP-uptake, and suggest that cAMP could function as a secreted messenger from the neural retina to the RPE.

## Results

**Figure 1:**
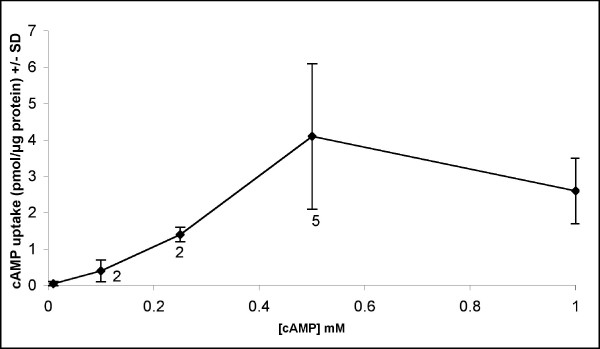
**cAMP uptake in isolated RPE**. Uptake of cAMP by isolated RPE following 30 min treatment with varying concentrations of exogenous cAMP. n = 4 except where noted.

RPE was isolated from dark-adapted fish and incubated in LCHER, a modified Ringer's solution. To test whether cAMP was taken up by isolated RPE, we used ^3^H-cAMP to test directly for import. We found that cAMP was indeed taken up and that cAMP uptake was saturable (see Figure 1). Comparisons of the amount of cAMP taken up revealed statistically significant differences among the concentrations.

To determine whether exogenously applied cAMP could induce pigment granule aggregation, isolated RPE was incubated for 30 minutes and then treated with increasing concentrations of cAMP for an additional 30 minutes. To assess the affects of these manipulations, cells were fixed and their pigment indices calculated. Pigment indices are the ratio of the length of the cell occupied by pigment and the total length of the cell. After the first 30 minute incubation, t_0 _samples had pigment indices reflective of a highly dispersed status (PI = 0.95 ± 0.02, n = 7). Pigment granule aggregation was observed in RPE after incubating for 30 minutes in cAMP; the extent of aggregation was a function of the dose applied. Maximal aggregation was attained by treatments as low as 1.0 mM cAMP (PI = 0.67 ± 0.02, n = 7); cells treated using higher concentrations of cAMP were not statistically significantly more aggregated. Therefore, a concentration of 1.0 mM cAMP was used in subsequent experiments that required induction of pigment granule aggregation.

**Figure 2:**
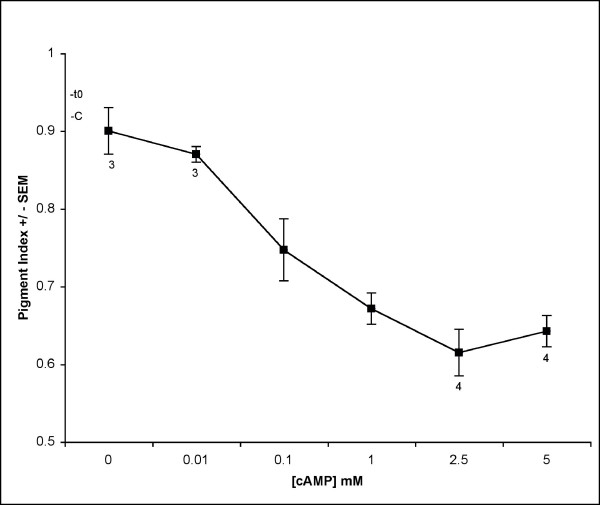
**Dose response analysis of cyclic AMP's effects on pigment granule aggregation in isolated RPE cells**. The t_0 _PI was 0.95 ± 0.02 (n = 7) after a 30 minute preculture. RPE cells were cultured in cAMP for 30 minutes. The control PI was 0.91 ± 0.02 n = 7, except where noted.

**Figure 3:**
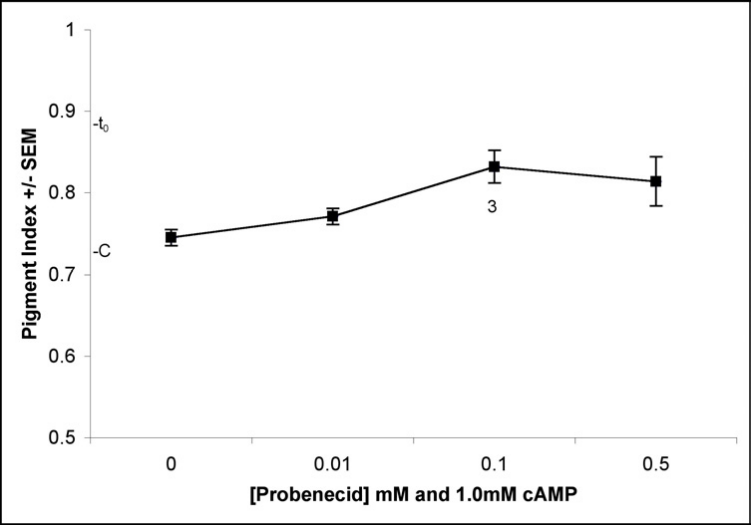
**Effects of probenecid on cAMP-induced aggregation**. The t_0 _pigment index was 0.88 ± 0.03 (n = 4) after a 30 minute preculture. The control, containing 1.0 mM cAMP and DMSO, had a pigment index of 0.72 ± 0.03 (n = 4, except where noted).

**Figure 4:**
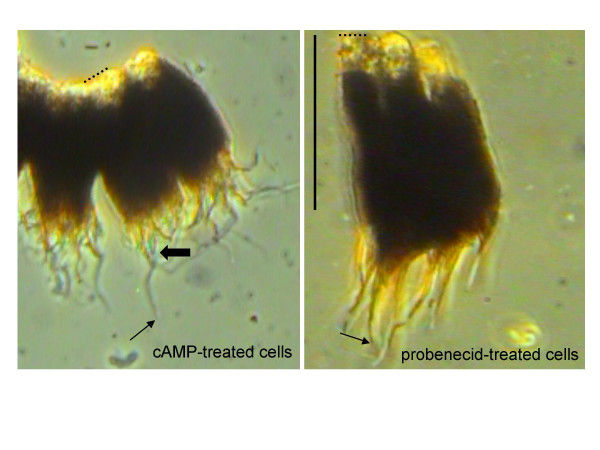
**RPE cells with pigment aggregated and dispersed**. Phase contrast images illustrate RPE with pigment granules aggregated in response to treatment with 1 mM cAMP (left) and RPE with pigment granules dispersed as a consequence of inhibition of cAMP-induced aggregation by probenecid (0.5 mM; right). Dotted lines indicate the position of the base of the cell, and the thin, diagonal arrow indicates the position of the end of the longest apical process for the same cell. For the cAMP-treated cells, a thick, horizontal arrow indicates the position of the distal-most pigment granule; for the probenecid-treated cells, that position corresponds with the end of the process. Scale bar equals 50 μm.

To test whether cAMP-induced aggregation was a consequence of uptake, we tested whether it could be blocked by an organic anion transport inhibitor. The experiments that involved inhibition of organic anion transport systems with probenecid were performed in the presence of 1.0 mM cAMP. Probenecid, a known organic anion transport system inhibitor [14], was found to inhibit cAMP's ability to induce pigment granule aggregation. A dose response analysis of the effect of probenecid on cAMP-induced pigment granule aggregation demonstrated that probenecid blocked pigment granule aggregation induced by 1.0 mM cAMP in a dose-dependent manner (see Figure 2). When probenecid was introduced to the RPE immediately prior to the introduction of cAMP, the degree of pigment granule aggregation was reduced at all of the doses tested (see Figure 3). The 0.1 mM probenecid dose was maximally effective at inhibiting pigment granule aggregation (PI = 0.83 ± 0.02, n = 3); the pigment index at a higher dose of 0.5 mM probenecid (PI = 0.81 ± 0.03, n = 4) was not significantly different. Images of cells treated with cAMP alone or with cAMP in the presence of probenecid can be seen in Figure 4.

## Discussion

The experimental results reported here suggest that bluegill is equipped with an organic anion transporter which imports cAMP in saturable manner. Cyclic AMP uptake results in pigment aggregation, and inhibition of uptake blocks cAMP-induced aggregation. We infer that organic anion transport systems provide the route by which cAMP crosses RPE cell membranes since probenecid inhibits extracellular cAMP from causing aggregation. A great deal of experimental evidence shows probenecid specifically inhibits organic anion transport, and it acts at several sites. For example, probenecid (0.1 mM) has been shown to inhibit the transport of fluorescein across RPE cell membranes [14]. Probenecid (1.0 mM) has been shown to inhibit the efflux of cAMP synthesized intracellularly in a number of tissues (see [15]), including human fibroblasts and turkey red blood cells [16], and in rat glioma cells without altering the intracellular synthesis of cAMP [17]. Its actions target a variety of organic transporters of the OAT family (see [18]) which in some cases seem more closely associated with import activities, and members of the multidrug resistance protein (MRP) family, including MRP4 and MRP5, whose activities have been linked to export of cyclic nucleotides (see [19]).

Cyclic AMP uptake has been demonstrated in liver and kidney cells [16], but this is the first demonstration of cAMP uptake in RPE. Organic anion transport systems may be the means by which uptake occurs [16]. Multidrug resistance proteins (MRP) 4 and 5 have been shown to mediate cyclic nucleotide efflux in a variety of cell types (reviewed in [15]), but to date has not been shown to be expressed in retina. Cyclic AMP secretion by *Dictyostelium discoideum *is known to be directly related to intracellular levels of cAMP [20]. Secretion of cAMP by the neural retina would increase the concentration of cAMP in the subretinal space and could result in cellular uptake of cAMP. Since stimulation of adenylyl cyclase by cAMP has not been observed in metazoans and since adenosine and ATP failed to induce pigment granule aggregation [8], it seems possible that cAMP located in the subretinal space could be taken up by RPE cells and could then stimulate pigment granule aggregation by activating cAMP-dependent protein kinase. Such an uptake mechanism may be the principal mechanism by which the neural retina signals darkness to the RPE of fishes.

Extracellular cAMP that enters RPE cells via organic anion transport systems induces pigment granule aggregation. According to the model proposed by García and Burnside [8], the neural retina produces the cAMP that induces pigment granule aggregation. Many neurochemical agents associated in other systems with increases in cAMP have been shown to have little or no effect on pigment granule aggregation in RPE isolated from green sunfish. For example, adenosine receptors positively linked to adenylyl cyclase are expressed by human RPE [21], yet adenosine failed to induce pigment granule aggregation in RPE isolated from green sunfish [8]. Therefore, if a receptor-mediated system coupled to adenylyl cyclase is employed in mediating the response of the RPE cells to exogenously applied cAMP, the receptors must be specific for cAMP since other adenine nucleosides do not induce aggregation [8].

The signal for RPE to disperse pigment granules in response to light is likely to vary among species of fishes. In species, such as green sunfish, in which pigment granule dispersion in the RPE is initiated upon light onset and shows no circadian rhythmicity [22], pigment granule dispersion could result from a combination of effects including cAMP efflux and/or degradation. This inference is based on the premise that cyclic nucleotide levels in the photoreceptors remain elevated until light onset, and on the presumption that cAMP efflux from the photoreceptors provides the primary signal for dark adaptation in the retina. In fishes whose RPE disperse pigment granules after the onset of light, depletion of cAMP in the subretinal space and then in the RPE may be sufficient to drive pigment granule dispersion. However, in fishes in which pigment dispersion anticipates light onset, it seems more likely that a neurotransmitter or neuromodulator, such as dopamine, is involved [23]. In this case, the extracellular signal could act to either lower intracellular cAMP or override its effects via another second messenger pathway. Evidence from John and Gring [24], suggests that bluegill RPE disperses pigment granules prior to light onset, increasing the likelihood that some mechanism besides cAMP efflux or degradation is involved in generating the light-adaptive response.

## Conclusion

In conclusion, the studies reported here suggest that RPE isolated from bluegill takes up cAMP in a saturable manner, that cAMP induces pigment granule aggregation in retinal pigment epithelium isolated from bluegill, and aggregation induced by cAMP treatment is inhibited by treatment with probenecid, an organic anion transport inhibitor. A model for cAMP acting as a cell-to-cell messenger to signal darkness to the RPE is supported by these studies. Furthermore, these studies raise the possibility that cAMP depletion in the RPE could by itself be sufficient to serve as a light signal in some species of fish.

## Methods

### Experimental animals

Wild-caught bluegill (*Lepomis macrochirus*) were procured from the San Marcos River Cummings dam (San Marcos, TX) and from Canyon Lake (TX) and were collected by electrofishing methods. Bluegill raised on a farm in Arkansas were purchased from Johnson Lake Management (San Marcos, TX). The fish ranged in size from 6 to 15 cm in length, and no more than 20 fish were kept in one tank at a time. Fish were housed in 55-gallon aquaria filled with tap water and aerated. Fish were maintained in such aquaria for a minimum of two weeks before dissection to allow the fish to become acclimated to a light cycle of 14 hours light/10 hours dark for the cAMP and probenecid dose response experiments with light onset occurring at 7 a.m.

### Solutions

HEPES-buffered, low calcium Ringers solution (LCHER) prepared on the day of each experiment for use in cAMP and probenecid dose-response analyses (see below). LCHER contained 24 mM NaHCO_3_, 116 mM NaCl, 5 mM KCl, 1 mM NaH_2_PO_4_, 26 mM dextrose, 4 mM HEPES, 1 mM ascorbic acid, 0.8 mM MgSO_4_, 1 mM EGTA and 0.9 mM CaCl_2_, titrated to a pH of 7.2. Low calcium (<10^-5 ^M free Ca^2+^) LCHER was used instead of normal LCHER (1.8 mM Ca^2+^) because low levels of calcium had been reported to be permissive for cAMP-induced pigment granule aggregation [7]. Cyclic AMP (10 mM; Boehringer-Mannheim, Indianapolis, IN) was prepared on the day of each experiment, and serial dilutions were prepared from this stock. Serial dilutions of probenecid (Sigma, St. Louis, MO) were made from a stock solution (10 mM) prepared in dimethylsulfoxide (DMSO; Sigma).

### Isolation of RPE sheets

RPE tissue was isolated from *L. macrochirus *for all experiments in this study. To facilitate separation of RPE from retina, fish were dark-adapted in aerated aquaria for 20 to 45 minutes immediately before dissection. Dissections done after 20 minutes and before 40 minutes of dark adaptation resulted in RPE sheets with few or no rods and cones fragments present with the tissue. After dark adaptation, the fish were killed by spinal section and pithing in total darkness. The remainder of the dissection was carried out under dim, incandescent light. After pithing, the eyes were enucleated and hemisected. The eyecup was flushed with LCHER from a pasteur pipet to dislodge RPE sheets. The RPE sheets were flushed from both eyes into a large weighing boat or a 100 mm petri dish lined with parafilm. The RPE sheets were then lifted with a modified large bore micropipet tip fitted with a latex pipet bulb and placed into the appropriate culture or treatment vessels. RPE sheets were divided into six portions: one portion (t_0 _sample) into a 1.5 ml microfuge tube and the other five portions (one control and four treatments) into a 24 well plate. RPE from different fish were not pooled during treatments. All experiments were conducted between 12:00 and 6:00 p.m. at approximately 25°C.

### Treatment and fixation of RPE sheets

RPE sheets were incubated in LCHER in 24 well plates on a gyratory shaker (<50 rpm). Drugs were added to incubating tissue from 10× stock solutions after a 30 minute incubation period that allowed unsolicited pigment granule dispersion [8]. A t_0 _sample was fixed immediately prior to the addition of drugs. Drug treatments lasted 30 minutes. After treatment 5× fix was introduced to the samples, yielding final concentrations of 0.8% w/v K_3_Fe(CN)_6_, 0.5% w/v paraformaldehyde (EM Science), and 0.5% v/v glutaraldehyde (EM Science). Fixative was prepared in phosphate buffered saline on the day of use.

### cAMP uptake

RPE sheets incubated in LCHER for 15 to 30 minutes were then placed in microcentrifuge tubes containing 1 μCi/ml ^3^H-cAMP along with increasing concentrations of unlabeled cAMP. The tissue was incubated for 30 minutes on a gyratory shaker (<50 rpm). The caps of the microcentrifuge tubes were left open to permit gas exchange. The incubation medium was then removed, and RPE sheets were rinsed three times with ice-cold PBS buffer. RPE sheets were homogenized in 100 μl distilled water, using disposable pestles. Homogenates were then centrifuged at 800 × *g *for 10 minutes to remove pigment granules, and the supernatants were pipetted into numbered eppendorf tubes. The pellets were resuspended in 100 μl distilled water and centrifuged again at 800 *X *G for 10 minutes. The supernatants were combined in eppendorf tubes with supernatants from the first centrigugation. Supernatants were used both for protein assay using the Bradford method and scintillation counting.

### Quantification of pigment position and statistical analysis

The RPE pigment index was determined 24–72 hours after fixation using a Zeiss photomicroscope equipped with phase contrast optics. Cells were viewed at 500× magnification. The RPE sheets were placed on a slide and minced with a razor blade or the edge of a coverslip, and a wet mount was prepared. Individual cells and cells from clusters that had both their apical and basal ends visible were measured along their apico-basal axes. The ratio of the distance from the base of the cell to the furthest out pigment granule to the total cell length was recorded as the pigment index (PI). A PI > 0.85 is dispersed, while a PI < 0.75 is aggregated. To be measured, the cells had to meet the requirements of (1) possessing at least three visible processes and (2) being greater than 50 μm in length. Twenty-five to thirty cells were measured for each sample, and the mean PI was determined.

Dose response curves were plotted to determine if cAMP and/or probenecid treatment affected the mean PI values. Error bars represent ± standard errors of mean (SEM). PIs plotted in the graph are mean PIs of all the samples used in the each study. "N" represents the number of samples measured. Statistical significance among experimental treatments were determined using single factor analysis of variance (ANOVA) and Tukey's HSD posthoc analyses. All statistical analyses were performed using S-PLUS 6.1. A P value of < 0.05 was assumed to indicate significant difference.

## Abbreviations

ANOVA Analysis of Variance

DMSO dimethylsulfoxide

cAMP Cyclic Adenosine monophosphate

EGTA Ethyleneglycotetraacetic acid

HEPES 4-(2-hydroxyethyl)-1-piperazineethanesulfonic acid

LCHER HEPES-buffered, low calcium Ringers solution

PI Pigment Index

rpm rotations per minute

RPE Retinal Pigment Epithelium

SEM Standard Error of Mean

## Authors' contributions

TAK conducted all the pharmacological experiments and prepared the first draft of the manuscript. VR carried out statistical analyses of the raw data, prepared the graphs and formatted the first draft. SM performed the cAMP uptake studies. DMG conceived of the study, participated in its design, oversaw the entire project, and finalized the manuscript for publication. All authors read and approved the final manuscript.
